# DNA Methylation Analysis of Turner Syndrome BAV

**DOI:** 10.3389/fgene.2022.872750

**Published:** 2022-05-31

**Authors:** Jacob Gutierrez, Brett A. Davis, Kimberly A. Nevonen, Samantha Ward, Lucia Carbone, Cheryl L. Maslen

**Affiliations:** ^1^ Department of Medical Informatics and Clinical Epidemiology, Oregon Health and Science University, Portland, OR, United States; ^2^ Department of Medicine, Oregon Health and Science University, Portland, OR, United States; ^3^ Department of Molecular and Medical Genetics, Oregon Health and Science University, Portland, OR, United States; ^4^ Division of Genetics, Oregon National Primate Research Center, Beaverton, OR, United States; ^5^ Knight Cardiovascular Institute, Oregon Health and Science University, Portland, OR, United States

**Keywords:** monosomy X, DNA methylation analysis, congenital heart defects, bisulfide sequencing, aortopathy

## Abstract

Turner Syndrome (TS) is a rare cytogenetic disorder caused by the complete loss or structural variation of the second sex chromosome. The most common cause of early mortality in TS results from a high incidence of left-sided congenital heart defects, including bicuspid aortic valve (BAV), which occurs in about 30% of individuals with TS. BAV is also the most common congenital heart defect in the general population with a prevalence of 0.5–2%, with males being three-times more likely to have a BAV than females. TS is associated with genome-wide hypomethylation when compared to karyotypically normal males and females. Alterations in DNA methylation in primary aortic tissue are associated with BAV in euploid individuals. Here we show significant differences in DNA methylation patterns associated with BAV in TS found in peripheral blood by comparing TS BAV (*n* = 12), TS TAV (*n* = 13), and non-syndromic BAV (*n* = 6). When comparing TS with BAV to TS with no heart defects we identified a differentially methylated region encompassing the BAV-associated gene *MYRF*, and enrichment for binding sites of two known transcription factor contributors to BAV. When comparing TS with BAV to euploid women with BAV, we found significant overlapping enrichment for ChIP-seq transcription factor targets including genes in the *NOTCH1* pathway, known for involvement in the etiology of non-syndromic BAV, and other genes that are essential regulators of heart valve development. Overall, these findings suggest that altered DNA methylation affecting key aortic valve development genes contributes to the greatly increased risk for BAV in TS.

## Introduction

Turner syndrome (TS) is a rare cytogenetic disorder caused by the partial or complete loss of a second sex chromosome, which occurs in 1 in 2,000 female live births (Shankar and Backeljauw, 2018). Girls with TS show a variety of clinical manifestations including short stature, premature ovarian failure, webbed neck, specific cognitive/visual spatial disabilities, hearing loss, thyroid dysfunction, scoliosis, endocrine disorders, autoimmune disorders, and cardiovascular disease. The most common cause of early mortality in TS is due to congenital heart defects, where patients with the most common 45, X karyotype have the highest burden of congenital defects and negative outcomes (Barr and Oman-Ganes, 2002). In addition to the increased post-natal cardiovascular defect related mortality risk, it is thought that over 99% of 45, X embryos are lost *in utero* with an increased prevalence for left-sided obstructive lesions otherwise known as Left Sided Heart Lesions (LSHL) (Barr and Oman-Ganes, 2002; Surerus et al., 2003; Urbach and Benvenisty, 2009).

Bicuspid Aortic Valve (BAV) is the most common congenital heart defect in the general population with a prevalence of 0.5%–2% (Giusti et al., 2017). BAV is defined as an aortic valve that consists of two leaflets as opposed to the normal three leaflet configuration of the Tricuspid Aortic Valve (TAV). BAV is considered to be a mild form of LSHL and is largely compatible with life, leading to the relatively high prevalence in the general population (Parker and Landstrom, 2021). The specific negative cardiovascular outcomes of BAV include valve calcification, stenosis, aortic endocarditis, aortic dilation, and aortic aneurysm; collectively known as aortopathy. Approximately 40% of patients with BAV go on to develop some form of aortopathy in their lifetime (Liu et al., 2019). TS patients with the 45, X karyotype have the highest burden of BAV with a prevalence around 30% with near complete penetrance of developing aortopathy (Miller et al., 1983). There is a significant sex bias within BAV, where males account for approximately 75% of all BAV cases (Liu et al., 2019). The high incidence of BAV in TS females and the bias towards karyotypically normal 46, XY males suggests that having one X chromosome predisposes individuals to the development of BAV and BAV associated aortopathy.

Despite the high prevalence in the general population, most of the etiology of BAV is not known. However, a genetic component of BAV has been identified as 10%–40% of BAV is familial (Silberbach, 2009). Mutations in *NOTCH1*, *GATA5*, *NKX2*.5, and *ROBO4* are known to cause BAV in some families, but the majority of BAV cases are simplex and of unknown etiology (McKellar et al., 2007; Qu et al., 2014; Shi et al., 2014; Gould et al., 2019). In the case of BAV in TS, a recent whole exome sequencing study has identified copy number variation of the X chromosome escape gene *TIMP1* coupled with functional linked SNPs in *TIMP3* to be associated with BAV and aortopathy in TS subjects with exome-wide significance (Corbitt et al., 2018). Although these genes show a very significant association with BAV, *TIMP1/3* deficiency only explains roughly 20% of the occurrence of BAV in TS. Taken together, these studies have shown that BAV is a complex and genetically heterogeneous condition.

DNA methylation (DNAm) alterations associated with BAV have been detected in primary aortic wall tissue and within the aortic valve itself in addition to non-coding RNA expression differences detectable in blood samples of BAV subjects (Pan et al., 2017; Björck et al., 2018; Pulignani et al., 2019). DNAm analysis of TS has identified genome-wide hypomethylation when compared to healthy 46, XX females and 46, XY males (Sharma et al., 2015; Trolle et al., 2016). Together, these findings suggest a role for epigenetic regulation both in TS and BAV that have not been explored. This study aims to address this gap by identifying DNAm alterations associated with TS BAV as well as between TS and euploid females with BAV to detect possible epigenetic modifications in BAV-associated genes and pathways that may further explain the high incidence of BAV and aortopathy in TS.

## Methods

### Samples and Study Design

All blood samples were collected by the GenTAC consortium and supplied through the NIH-sponsored BioLINCC biorepository (Kroner et al., 2011). In order to control for known sources of variation that could confound DNAm studies, all samples included were of non-smoking individuals. Smoking status was determined by subject self-reporting at time of GenTAC enrollment. For TS subjects, karyotype and BAV status was primarily determined based on clinical information gathered at GenTAC enrollment. A subset of subjects had karyotype information confirmed *via* molecular karyotyping performed in a previous exome sequencing study (Corbitt et al., 2018). All subjects were over 13 years of age in order to minimize adolescent age effects and both biological groups displayed large overlap in age ranges (Table 1). Enrollment and studies have Internal Review Board approval and all study subjects had informed consent for participation. For subjects under the age of 18 years a child assent form was completed in addition to the informed consent signed by a legal guardian. A total of 36 whole blood DNA samples from three groups (TS with a confirmed BAV, TS BAV; TS with a confirmed tricuspid aortic valve, TS TAV; and euploid females with a simplex BAV, 46, XX BAV) were analyzed using targeted methylation sequencing. Three samples did not yield enough reads to be included in downstream analysis following read deduplication. Unexpectedly, two TS samples showed X chromosome methylation levels comparable with the 46, XX BAV samples indicating mosaicism of the X chromosome. The newly developed DAMEfindeR allelic methylation analysis method was used to confirm X inactivation within these samples leading to their exclusion (Orjuela et al., 2020). Following sample exclusion a total of 31 samples were used for differential methylation analysis.

**TABLE 1:** Study subject characteristics.

Study Groups (karyotype and aortic valve status)	Sample Size (*n*)	Average Age and Range (years)
46,XX BAV	6	52 (28–67)
45,X TAV	13	35 (44–68)
45,X BAV	12	42 (16–65)

### Targeted Methylation Sequencing

Genomic DNA samples were submitted for targeted methylation sequencing library preparation at the OHSU Epigenetics Core. The Illumina TruSeq-Methyl Capture EPIC Library Prep Kit (TruSeq-Methyl Capture EPIC, cat # FC-151-1002, Illumina Inc., San Diego, CA, United States) was used to prepare libraries, which interrogates the same genomic loci as the Illumina MethylationEPIC microarray. Briefly, 500–1,000 ng of high-quality DNA was fragmented using the Bioruptor Pico sonicator (Diagenode). The captured fragments were then bisulfite converted and amplified by PCR. Fragment size was analyzed *via* TapeStation (Agilent), quantified by Qubit (Invitrogen), and qPCR (KAPA) prior to sequencing. All sequencing was done at the OHSU Massively Parallel Sequencing Shared Resource using the NovaSeq 6000.

### Data Processing and Quality Control

Bisulfite sequencing data was aligned using the ENCODE WGBS standard (ENCODE consortium, 2021). Raw sequencing reads were assessed for quality with FastQC v0.11.9, adapter trimmed with TrimGalore v0.6.6, aligned to the hg38 assembly using Bismark v0.23.0 and deduplicated (Andrews, 2010; Krueger and Andrews, 2011). Bismark coverage reports were generated using BismarkMethylationExtractor command and processed in R v3.6.2 using MethylKit v1.12.0 (Akalin et al., 2012). CpG data was filtered for 10X coverage for each sample, CpGs with majority coverage within each study group was used for differential methylation analysis.

Due to some TS samples showing X chromosome features similar to euploid samples, X inactivation status for all samples was validated using the newly developed allelic methylation analysis tool DAMEfinder v1.2.0 (Orjuela et al., 2020). Briefly, MethTuple v1.5.3 was applied to the same aligned data to detect di-CpG methylation status within the same molecule (read) (Hickey, 2020). These di-CpG loci are then filtered for 10X coverage and loci with complete coverage across all available samples were retained. Following the original publication, mean allelic methylation scores from X chromosome gene promoters were extracted to distinguish samples that have bi-allelic methylation as a proxy for X inactivation.

Principal components analysis (PCA) was done using the R stats prcomp function. Principal Components Partial R Squared (PCPR2) analysis is an extension of PCA which allows for the assessment of technical factors across all principal components and was performed using a custom R function following the original publication (Fages et al., 2014).

### Differential Methylation Analysis

Surrogate Variable Analysis v3.34.0 from the sva R package was used to adjust the differential methylation model for batch effects and cell type heterogeneity, required for the analysis of DNAm in whole blood (Leek and Storey, 2007; Houseman et al., 2015). SVA models known batch effects such as the enrichment pool or sequencing run in addition to unknown sources of variation including cell type heterogeneity. SVA was selected to adjust for celltype composition due to stable performance across multiple studies, reference free nature, and application across multiple platforms (McGregor et al., 2016; Kaushal et al., 2017). DMRs were detected using a two-step approach with differentially methylated CpGs being detected using Limma v3.42.2 adjusted for Age and Surrogate Variables followed by DMR detection using Comb-P v33.1.1 using default parameters (Pedersen et al., 2012; Ritchie et al., 2015). The statistical significance threshold was set at <0.1 due to the hypothesis generating nature of this study. Significant DMRs were called with a sidak adjusted *p* value < 0.1 and no difference in methylation threshold was used due to the phenotype of interest, BAV, occurring early in development which may not lead to a large difference in methylation states between our groups of interest. Due to 46, XX karyotype samples being subject to X inactivation and being incomparable to a single activated X chromosome, the TS v. 46, XX comparison had CpGs on the sex chromosomes excluded from comb-p DMR detection.

Genes overlapping these DMRs were annotated using Genomation v1.18.0 with DMRs being annotated to genes overlapping exon, intron, or promoter regions being deemed genic and all other DMRs intergenic (Akalin et al., 2015). GeneHancer 2017 data was downloaded from GeneCards (Fishilevich et al., 2017). ENCODE cCRE regulatory regions were downloaded from the SCREEN ENCODE portal (The ENCODE Project Consortium Snyder et al., 2020). LOLA v1.16.0 was used to analyze enrichment for known genomic loci by comparing DMRs with their appropriate background regions to the available databases with cCRE and genehancer files processed into database collections for LOLA analysis using a custom script (Sheffield and Bock, 2016). TFBS motif enrichment was performed using HOMER v4.11.1 to analyze TF networks which could be altered by DNAm alterations. TFBS sequence logos were generated by using motifs files produced by HOMER and were visualized with ggseqlogo v0.1 (Wagih, 2017). DMRs were analyzed in bulk or subset by hypo/hyper methylation status, with background regions being defined as all tested regions extracted from Comb-P (Heinz et al., 2010). STRINGdb and ENRICHR were used to assess pathways contributing to the extracted gene lists (Chen et al., 2013; Szklarczyk et al., 2019). Reactome Pathway analysis was performed using web based Analysis Tools (Jassal et al., 2019). Plots were created using ggpubr v0.4.0 and ggplot2 v3.3.3 (Wickham, 2016; Kassambara, 2020).

## Results

### Blood From Turner Syndrome Bicuspid Aortic Valve Patients Does Not Show Global DNAm Differences When Compared to Turner Syndrome Tricuspid Aortic Valve

**FIGURE 1:**
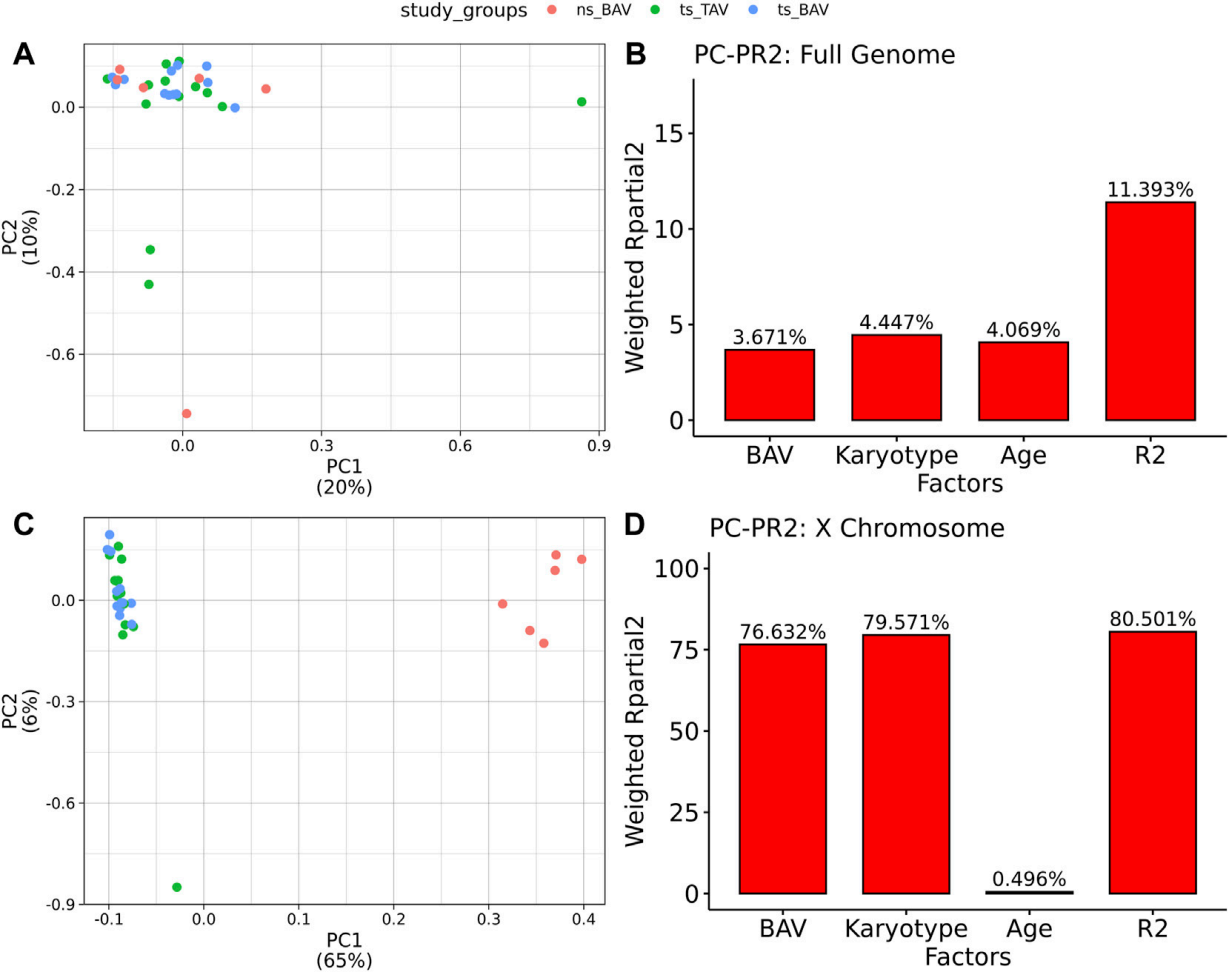
Principal Components Analysis of Methylation data indicates TS samples are distinct from ns BAV on the X chromosome. **(A)** PCA biplot of first 2 principal components for all autosomes. **(B)** PCPR2 analysis of biological variables for autosomes. **(C)** PCA biplot of X chromosome. **(D)** PCPR2 analysis of biological variables for the X chromosome. Note, PCPR2 is sensitive to multicollinearity and overestimates BAV contribution due to unbalanced representation for each karyotype.

All 31 samples analyzed in this study showed robust bisulfite conversion with <1% nonCpG methylation for all samples, with a mean alignment rate of 81%. After filtering for CpGs with at least 10X coverage, all samples yielded an average of 3.1M CpGs, with a mean read depth of 30X. Once CpGs were filtered for majority coverage across each study group, there were approximately 2.7M CpGs used for downstream analysis with a mean read coverage of 36X. PCA did not separate the samples by study group (Figure 1A), suggesting the absence of global DNAm differences and possibly the presence of high variability within each group. Such variability is expected due to the use of a cohort of human blood samples from a multi-site registry which could have differences in DNA extraction and storage. The contribution of both BAV and karyotype was inferred from Principal Component Partial R Squared (PC-PR2) analysis with the main variables of interest explaining roughly 11% of the variation (Figure 1B). PCA of the X chromosome CpGs shows clear separation based on karyotype (Figure 1C). Within the X chromosome, karyotype alone is the major contributor of the variation explaining roughly 80% of the variation (Figure 1D).

### Detected DNAm Alterations Between Turner Syndrome Bicuspid Aortic Valve vs. Turner Syndrome Tricuspid Aortic Valve Suggests Functional Differences

**FIGURE 2:**
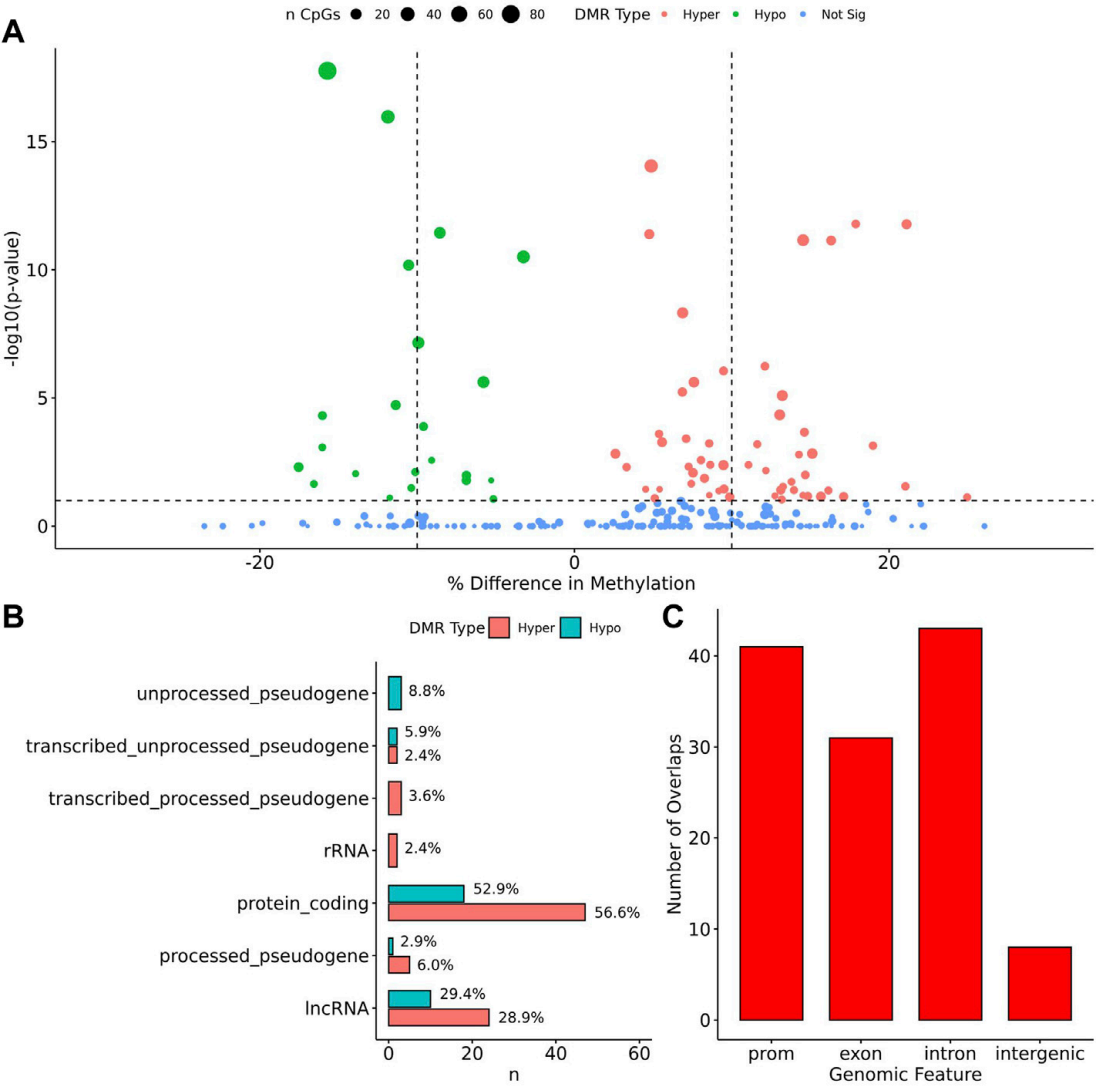
76 DMRs were detected in TS BAV mostly near protein coding regions. **(A)** Volcano plot of all regions detected colored by DMR type, hypermethylated, hypomethylated, or not significant with dashed lines at −log10 (0.1) and ±10% methylation difference. **(B)** Barplot of gene type annotations for all genes overlapping DMRs or the nearest gene when DMRs lack overlap. **(C)** Barplot of promoters, exon, intron, and intergenic regions overlapping DMRs.

When comparing TS BAV to TS TAV, a total of 76 significant DMRs (*q*-value < 0.1) were detected of which 44 showed a methylation difference >10% (Figure 2A). The detailed results for these DMR are shown in Supplementary Table 1. The majority of DMRs (*n* = 51; 71%) were found to be hypomethylated in TAV and largely overlapped with genes and lncRNAs (Figures 2B,C). However, gene set enrichment analysis and STRINGdb analysis did not detect any significant enrichment for biological function for genes overlapping DMRs (Chen et al., 2013; Szklarczyk et al., 2019).

**FIGURE 3:**
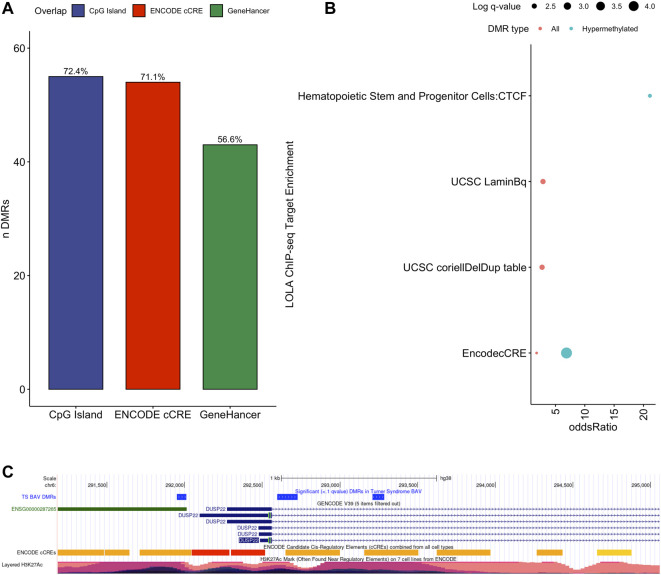
Significant LOLA genome feature overlap enrichment for TS DMRs (<0.1 *q* value). **(A)** Dotplot of LOLA enrichment for UCSC features, Cistrome epigenomics database, Sheffield and Bock (2016) DNAse database, and Encode cCRE/GeneHancer overlap. **(B)** Dotplot of LOLA enrichment for CODEX database ChIP-seq peak overlap for NOTCH1, MYH11, KDM4A, and HDAC2. **(C)** Three significant TS BAV DMRs displayed in the UCSC Genome Browser (http://genome.ucsc.edu) with ENCODE cCREs and H3K27ac tracks over the *DUSP22* gene locus.

To assess the possible function of the DMRs, we overlapped them with known regulatory enhancers from GeneHancer, ENCODE cCRE, and annotated CpG Islands (Fishilevich et al., 2017; The ENCODE Project Consortium Snyder et al., 2020; Karolchik et al., 2004). We found that the majority of DMRs overlap cCRE and CpG islands (72.4% and 71.1%, respectively) suggesting that these DMRs reside in functionally relevant regions of the genome (Figure 3A). To aid in the interpretation of these DMRs we sought to compare them to previously generated sequencing studies using locus overlap enrichment analysis (LOLA) which compares these DMRs to databases comprising of genomic regions to identify enrichment using Fisher’s exact test for features such as transcription factor binding sites from ENCODE, Cistrome database features, DNase hypersensitive sites from Sheffield et al. (2013), CODEX database features, UCSC browser features, and a custom database reflecting ENCODE cCRE elements. We then tested for LOLA enrichment among hypermethylated and hypomethylated DMRs and found that only hypermethylated DMRs displayed significant enrichment for cCRE (Figure 3B) (Sheffield and Bock, 2016). To investigate the functional relevance of these significantly enriched cCRE elements the genes associated with these regions were explored. We identified various genes associated with congenital heart defects (*DUSP22* and *MYOM2*) and another gene (*UTS2*) which is known to protein expression changes affecting cardiovascular function in patients with congenital heart defects (Simpson et al., 2006; Grunert et al., 2014; Thorsson et al., 2015; Auxerre-Planti é et al., 2020). Together these results suggest that hypermethylated cCRE elements may play a functional role in the development or pathology of BAV in TS. Next we sought to determine if genes whose promoters overlap with DMRs belonged to biologically relevant pathways, we performed Reactome pathway enrichment analysis and observed significant enrichment for plasma lipoprotein clearance (*NR1H2, ACAT2*) (FDR = 0.04), glucagon signaling in metabolic regulation (*GNAS*) (FDR = 0.04), and NR1H2 & NR1H3 regulate gene expression to limit cholesterol uptake (*NR1H2*) (FDR = 0.07) (Supplementary Table 2). Metabolic regulation and cholesterol regulation do not have well understood connections to BAV and LSHL, however these results suggest a potential link that could be further explored.

Investigating genes directly overlapping DMRs, notable findings on autosomes include *DUSP22* on chromosome 6 which shows significant differences in methylation along most of the locus covering three separate DMRs with a 7.5% average difference in methylation (Figure 3C). Other noteworthy genes include *MYRF* and *ATP11A* which reside in the intronic regions of these genes and overlap cCRE elements which may regulate the expression of these genes (Supplementary Figure 1). There was special interest for DMRS present on the X chromosome could shed light on X chromosome dynamics predisposing TS individuals to develop BAV and BAV associated aortopathy. Two DMRs were detected on the X chromosome, both of which overlapped CpG islands within pseudogenes *ANKRD11P2* and *FTH1P27*. No known regulatory elements were overlapping these X chromosome DMRs nor protein coding genes were found nearby from these DMRs.

To analyze potential transcription factor networks that could be altered by changes to DNAm within their binding sites, we assessed if the DMRs were enriched for known transcription factor binding site (TFBS) motifs using HOMER. Although no TFBS reached significance, we found that known regulators of heart valve development *PBX3* and *PKNOX1* were 15 and 19-fold enriched, respectively, which approached significance (*q* values = 0.1041) (Table 2). Specifically, these binding sites were present in three DMRs and their binding co-occurred with one another, which potentially suggests that their functions may be altered together by changes in DNAm. Moreover, the same three DMRs also overlapped cCRE regulatory elements, suggesting that DNAm alterations could produce functional differences in genes regulated by these elements. To explore this observation further, the nearest genes were extracted in order to investigate which pathways may be altered by changes in TF binding through DNAm alterations. Reactome pathway enrichment analysis for genes associated with PBX3/PKNOX1 motif cCRE’s revealed significant enrichment for signaling by hedgehog (*GNAS*) (FDR = 0.03), gene and protein expression by JAK-STAT signaling after interleukin-12 stimulation (*HNRNPF)* (FDR = 0.01)*,* and metabolism of angiotensinogen to angiotensin (*CTSZ*) (FDR = 0.05) (Supplementary Table 3). These pathways are all critical for the development and maintenance of the cardiovascular system which suggests that DNAm alterations may produce functional changes relevant to BAV and aortopathy. Returning to the TFBS motif enrichment analysis results, many well characterized “late” HOX gene TFBS motifs, known to contribute to limb and heart development, were also enriched within these DMRs that approached statistical significance (Table 2). Specifically, *HOXA10* was found to be enriched and is known to regulate heart development through interactions with *NKX2-5* with mutations in this gene known to cause BAV (Behrens et al., 2013). Presently the connection between “late” HOX genes and BAV development is not well understood, however when taken together these results indicate that DNAm alterations in these DMRs may produce functional changes in both genes and pathways which could lead to increased susceptibility to aortopathy development.

**TABLE 2:** Homer TFBS Motif enrichment results for all DMRs comparing TS BAV vs. TS TAV indicating *PBX3* and *PKNOX1* approach statistical significance (*q* value < 0.1).

All DMRs								
Motif	Name	*p*-value	*q* value-FDR	n Targets	% Targets	n Background	% Background	Fold enrichment
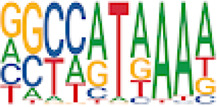	Hoxd11(homeobox) ChickenMSG-Hoxd11.Flag-ChIP-Seq (GSE86088)	1.00E-05	0.004	12	16.67	5.9	4.20	3.97
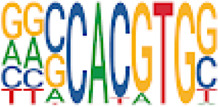	c-Myc (bHLH) mES-cMyc-ChIP-Seq (GSE11431)	1.00E-04	0.005	9	12.50	4	2.85	4.39
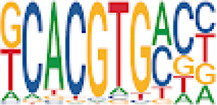	bHLHE41 (bHLH) proB-Bhlhe41-ChIP-Seq (GSE93764)	1.00E-03	0.028	24	33.33	22.1	15.88	2.1
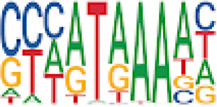	Hoxa13(homeobox) ChickenMSG-Hoxa13.Flag-ChIP-Seq (GSE86088)	1.00E-03	0.028	10	13.89	5.1	3.68	3.77
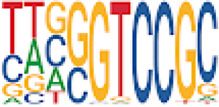	HINFP(Zf) K562-HINFP.eGFP-ChIP-Seq (Encode)	1.00E-03	0.039	15	20.83	12	8.60	2.42
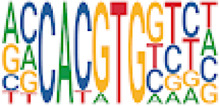	Max (bHLH) K562-Max-ChIP-Seq (GSE31477)	1.00E-03	0.068	11	15.28	7.8	5.57	2.74
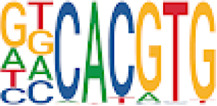	BMAL1 (bHLH) Liver-Bmal1-ChIP-Seq (GSE39860)	1.00E-02	0.104	19	26.39	18.7	13.42	1.97
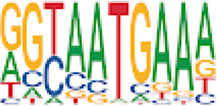	Hoxa10(homeobox) ChickenMSG-Hoxa10.Flag-ChIP-Seq (GSE86088)	1.00E-02	0.104	4	5.56	1.8	1.29	4.31
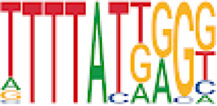	HOXB13(homeobox) ProstateTumor-HOXB13-ChIP-Seq (GSE56288)	1.00E-02	0.104	4	5.56	1.6	1.18	4.71
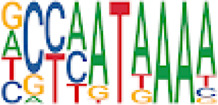	Hoxd13(homeobox) ChickenMSG-Hoxd13.Flag-ChIP-Seq (GSE86088)	1.00E-02	0.104	4	5.56	0.4	0.28	19.86
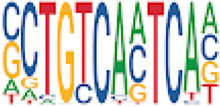	Pbx3(homeobox) GM12878-PBX3-ChIP-Seq (GSE32465)	1.00E-02	0.104	4	5.56	0.5	0.35	15.89
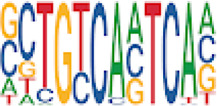	Pknox1(homeobox) ES-Prep1-ChIP-Seq (GSE63282)	1.00E-02	0.104	4	5.56	0	0.00	Inf

### Turner Syndrome Methylation Alterations Support Previous Findings

**FIGURE 4:**
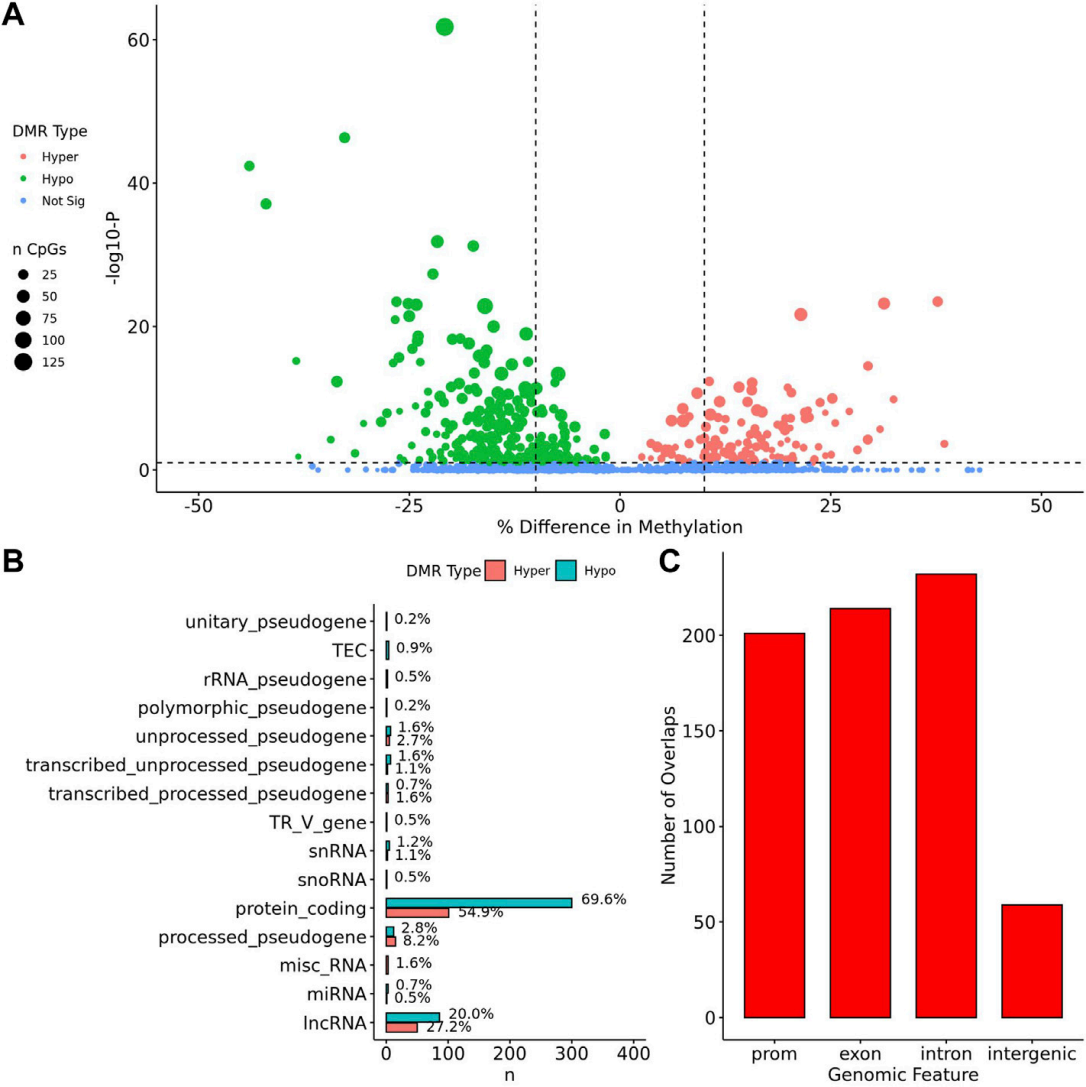
Feature enrichment for genes overlapping TS BAV DMRs. **(A)** Barplot of DMR overlap with CpG islands, ENCODE cCRE, and GeneHancer elements. **(B)** Dotplot of LOLA feature overlap with enrichment for cCREs, CTCF ChIP-seq peaks, Lamin B1 nuclear lamina interactions, and Coriell Cell Line Copy Number Variants (coriellDelDup table). **(C)** Three significant TS BAV DMRs displayed in the UCSC Genome Browser with ENCODE cCREs and H3K27Ac tracks over the DUSP22 gene locus.

Within the TS vs. 46, XX comparison a total of 414 DMRs (Supplemental Table 4) were detected with an adjusted *p* value < 0.1, of which 329 had a methylation difference >10% the majority of which (*n* = 281, 68%) are hypomethylated (Figure 4A). Similar to what was noted above, these DMRs largely overlap with protein coding genes and lncRNA (Figures 4B,C). Genes overlapping DMRs were annotated as previously done to be used for Geneset, Reactome pathway, and STRING enrichment analysis. Reactome pathway analysis revealed significant enrichment (FDR = 0.003) for activation of anterior *HOX* genes in hindbrain development during early embryogenesis due to the presence of four *HOX* genes within DMRs *HOXB3*, *HOXB6*, *HOXA3*, *HOXA4*, and *HOXC4*. Notably, *HOXA3* and *HOXB3* are known to contribute to cardiac development (Roux and Zaffran, 2016).

**FIGURE 5:**
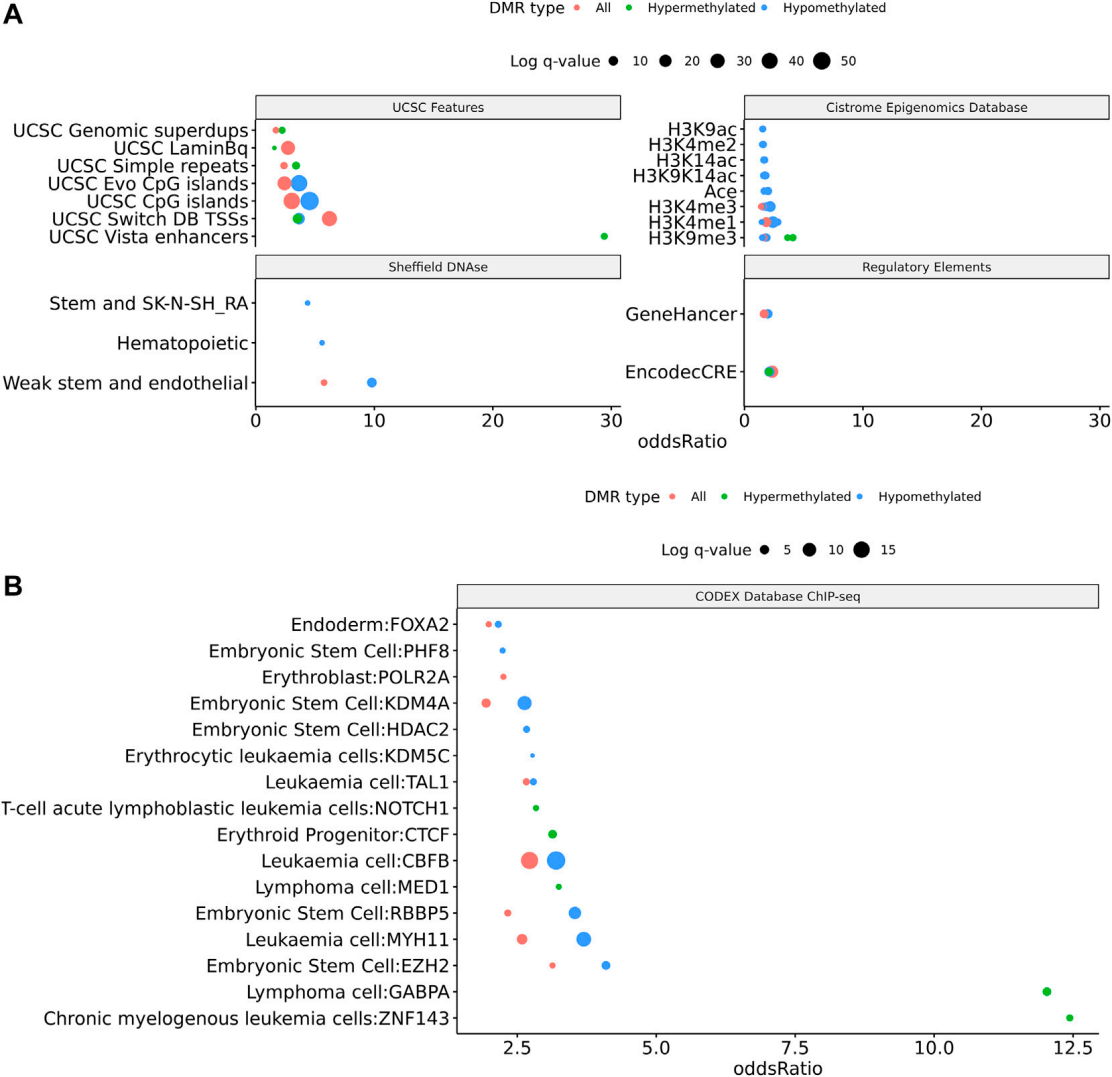
414 DMRs were detected in TS v. 46, XX which are largely hypomethylated and near protein coding regions. **(A)** Volcano plot of all regions detected colored by DMR type, hypermethylated, hypomethylated, or not significant with dashed lines at −log10 (0.1) and ±10% methylation difference. **(B)** Barplot of gene type annotations for all genes overlapping DMRs or the nearest gene when DMRs lack overlap.

Similar to the BAV comparison, cCRE and genehancer regulatory elements were overlapped to DMRs and LOLA analysis was used to augment interpretation of these gene regions by comparing them to previously generated datasets. DMRs were enriched for cCREs and only hypermethylated DMRs did not show enrichment for genehancer elements (Figure 5A). All DMRs subsets were enriched for cCREs and only hypermethylated DMRs did not show enrichment for genehancer elements (Figure 5A). The enrichment for these functional elements suggests that these DMRs may have functional roles at some stages of development. It was found TS DMRs show enrichment for CpG islands and evolutionarily conserved CpG islands identified by Cohen et al. (2011) (Figure 5A). These DMRs show enrichment for hematopoietic cells and weak stem-epithelial cell DNAse hypersensitivity sites derived from Sheffield et al., 2013 reflecting the use of blood DNA samples.

DMRs were significantly enriched for *ZNF143*, *GABPA*, *EZH2*, *RBBP5*, *HDAC2*, and *KDM4A* ChIP-seq binding sites (*q* values < 0.05) identified from the CODEX and ENCODE databases. All of these transcription factors are known epigenetic regulators of gene expression and development. In addition, there was significant enrichment for many known chromatin states such as heterochromatin (H3K9me3; assayed in prostate, breast, and hematopoietic stem cells) and promoters and primed enhancers (H3K4me3 and H3K4me1; assayed in prostate and breast) (*q* values < 0.05). In addition, there was significant enrichment for TFBS overlap for gene expression regulators including *SIN3A*, *CTCF*, *YY1*, and *POL2* (*q* values < 0.05) derived from ENCODE database (Supplementary Figure 2). DMRs show TFBS enrichment for *NOTCH1* (*q* value = 0.038) and the downstream *NOTCH* pathway gene *MYH11* (*q* values < 0.002) (Figure 5B). Mutations in *NOTCH1* are known to cause familial BAV disease, whereas mutations in *MYH11* cause hereditary thoracic aortic aneurysms (McKellar et al., 2007; Takeda et al., 2015; Kerstjens-Frederikse et al., 2016).

Homer TFBS motif enrichment was performed and it was found that all DMRs display enrichment for *PIT1* (*q* value = 0.00, a regulator of hormone expression), and *TBX20* approaching significance (*q* value = 0.15), a known regulator of heart development (Table 3) (Pfaffle et al., 1999; Kirk et al., 2007). Hypomethylated DMRs show enrichment for *TBX20* and *ZFX*. Interestingly, *ZFX* is an X escape gene that could contribute to the phenotypes seen in TS due to these individuals only having one X chromosome which has been previously detected to be differentially methylated in TS subjects (Trolle et al., 2016).

**TABLE 3:** HOMER TFBS motif enrichment results for all DMRs and hypomethylated DMRs for TS v. 46, XX indicating significant enrichment for *PIT1* and *ZFX* (*q* value < 0.1).

All DMRs								
Motif	Name	*p*-value	*q* value-FDR	*n* Targets	% Targets	*n* Background	% Background	Fold enrichment
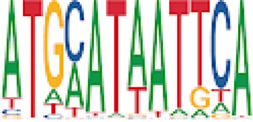	Pit1+1bp (homeobox) GCrat-Pit1-ChIP-Seq (GSE58009)	1.00E-04	0.026	7	1.79%	2.5	0.30%	5.97
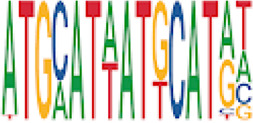	OCT:OCT-short (POU,Homeobox) NPC-OCT6-ChIP-Seq (GSE43916)	1.00E-03	0.139	11	2.82%	7.8	0.95%	2.97
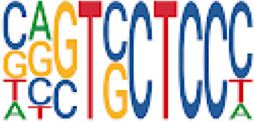	Znf263(Zf) K562-Znf263-ChIP-Seq (GSE31477)	1.00E-03	0.139	106	27.18%	167.2	20.34%	1.34
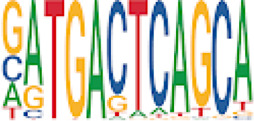	NF-E2 (bZIP) K562-NFE2-ChIP-Seq (GSE31477)	1.00E-02	0.158	4	1.03%	1.8	0.22%	4.68
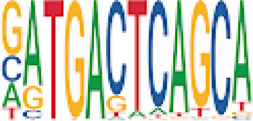	Tbx20 (T-box) Heart-Tbx20-ChIP-Seq (GSE29636)	1.00E-02	0.158	12	3.08%	10	1.21%	2.55
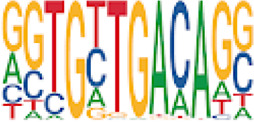	Pknox1(homeobox) ES-Prep1-ChIP-Seq (GSE63282)	1.00E-02	0.158	11	2.82%	8.9	1.09%	2.59
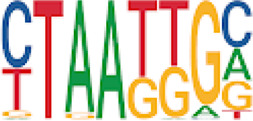	Isl1(homeobox) Neuron-Isl1-ChIP-Seq (GSE31456)	1.00E-02	0.158	49	12.56%	67.2	8.17%	1.54
Hypomethylated DMRs								
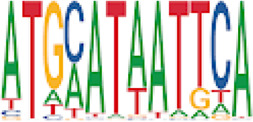	Pit1+1bp (homeobox) GCrat-Pit1-ChIP-Seq (GSE58009)	1.00E-06	0.001	6	2.76%	1.7	0.17%	16.24
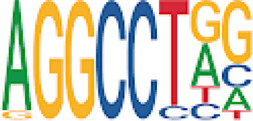	ZFX (Zf) mES-Zfx-ChIP-Seq (GSE11431)	1.00E-04	0.007	68	31.34%	189	19.77%	1.59
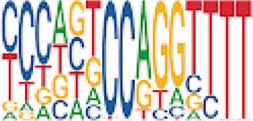	PRDM15 (Zf) ESC-Prdm15-ChIP-Seq (GSE73694)	1.00E-02	0.154	29	13.36%	69.5	7.27%	1.84

## Discussion

TS individuals are at a 60-fold increased risk of BAV compared to the general population. Although BAV is the most common congenital heart defect, there is little understanding of the epigenetic alterations associated with this condition in the general population let alone in TS individuals. Considering BAV is a developmental disorder, DNAm alterations might provide an insight into the genes or pathways that contribute to this condition. In this study we compared DNAm in blood obtained from 45, X TS individuals with BAV against TS individuals who have the normal tricuspid aortic valve to determine if differential DNA methylation could contribute to BAV etiology in TS. In addition, we compared individuals with TS and BAV to euploid women with BAV to see if DNA methylation alterations due to monosomy X correlates with the significantly increased risk for BAV in TS compared to having two X chromosomes.

We found significant DNAm differences between BAV and TAV in TS and observed that most of these DMRs overlap regulatory elements. Interestingly, when looking for TFBS motif enrichment we observed that *PBX3*, known to contribute to BAV in mice models through interactions with the chromatin remodeling complex MEIS1, approached significance (Stankunas et al., 2008). The regulator of *PBX*-*MEIS* interactions, *PKNOX1,* was also found to have motifs the same DMRs suggesting possible co-regulation of nearby genes during development (Schulte and Geerts, 2019).

Notable genes of relevance on autosomes include *DUSP22* on chromosome 6, which is known to activate JNK signaling in T cells and aged knockout mice show increased autoimmunity implicating immune system response. Additionally, a CNV in this gene has been linked to cardiac atrial septal defects suggesting this gene is important to the development and maintenance of the cardiovascular system (Li et al., 2014; Thorsson et al., 2015). We identified DMRs overlapping *MYRF* and *ATP11A* which are both genes directly associated with congenital heart disease including BAV (Rossetti et al., 2019; Szumska et al., 2019). In humans, *MYRF* is a key regulator of myelin development and is required for biosynthesis of oligodendrocytes; mutations in this gene are associated with a newly identified disorder, cardiac-urogenital syndrome, which is characterized by congenital heart defects including BAV (Bujalka et al., 2013; Rossetti et al., 2019). Mutations in this gene are also associated with non-myelin disease and orthologs play important roles in organisms without myelin such as *C. elegans*. Overall, these observations support an important role for *MYRF* during development which have not yet been fully explored (An et al., 2020). ATP11A is a ubiquitously expressed phospholipid flippase which could be important for cell-cell signaling through the cell membrane (Takatsu et al., 2014; Miyano et al., 2016; Segawa et al., 2016; Hawkey-Noble et al., 2020). The Deciphering Mechanisms of Developmental Disorders (DMDD) project conducted a mouse knockout screen to identify genes which confer embryonic lethality and found that *ATP11A* knockout mice had aortic defects indicating this gene is critical for normal heart development (Szumska et al., 2019). These two genes are interesting candidates for further analysis because they both have been independently associated with non-syndromic BAV and likely act outside of known pathogenic mechanisms of BAV development such as through *NOTCH* signaling (Szumska et al., 2019). Taken together, these findings support the hypothesis that in TS DNAm alterations might occur in genes and pathways relevant to BAV etiology.

An unexpected finding was enrichment for cholesterol biosynthesis and regulation within BAV DMRs. Studies of BAV in the euploid population have linked increased cholesterol levels in BAV patients with aortic stenosis and linear correlation of low-density lipoprotein levels and ascending aorta diameter (Alegret et al., 2015; Endo et al., 2015). BAV patients undergoing statin treatment were observed to have reduced progression of aortopathy following heart surgery, which was not found in the TAV counterparts (Taylor et al., 2016; Sequeira Gross et al., 2018). An interesting connection can be found when we consider that TS individuals are at an increased risk for dyslipidemia which presents at an early age, although cholesterol levels improve following hormone replacement therapy (Ross et al., 1995; Mavinkurve and O'Gorman, 2015). These findings suggest that there might be an unexplored connection between BAV, cholesterol regulation, and aortopathy which may contribute to TS BAV.

There were significantly more DMRs found when comparing TS BAV to 46, XX BAV subjects than the TS BAV to TS TAV comparison which is consistent with a larger impact of X chromosome monosomy on the epigenetic landscape. Over 99% of embryos with 45, X karyotype are not viable during development with most of these fetuses failing *in utero* due to LSHL. Therefore it would not be expected that DNAm alterations on the same scale as monosomy X, genome-wide hypomethylation, to be found in the TS BAV subjects who are compatible with life (Urbach and Benvenisty, 2009; Mortensen et al., 2012). Similar to previous findings, TS DMRs are hypomethylated across the full genome indicating that loss of a second sex chromosome leads to global changes to DNAm and potentially other epigenetic regulators which may predispose TS individuals to CHD during development.

An interesting result from this analysis has detected multiple HOX genes, critical for embryogenesis and hindbrain development, were found to be differentially methylated in TS compared to euploid women. This result is consistent with previous analyses of TS methylation patterns which found *HOXA4* and *HOXB6* to be hypermethylated compared to female controls (Sharma et al., 2015). Additionally, a rare copy number variant within the *HOXA* cassette has been identified to contribute to cases of LSHL in TS (Prakash et al., 2016). Together, these results suggests that dysregulation of *HOX* gene function may contribute to the greatly increased incidence of LSHL in TS. However, we cannot rule out the possibility that this methylation difference is broadly related to TS as opposed to being TS BAV-specific. Considering that *HOX* gene function is critical for the normal development of various body and organ systems, the dysfunction of this key developmental regulatory system may also contribute to the other phenotypes seen in TS which have yet to be fully explored.

We have found that TS DMRs show significant overlap with genomic targets for *NOTCH1*, and the downstream *NOTCH* pathway gene *MYH11*. *NOTCH1* mutations are known to cause familial BAV (McKellar et al., 2007). However, *MYH11* mutations are known to cause familial thoracic aortic aneurysms (Takeda et al., 2015). Vascular smooth muscle cells (VSMC) derived from induced pluripotent stem cells from BAV subjects implicate *NOTCH1* and *MYH11* expression in VSMC differentiation in aortopathy (Harrison et al., 2019). Together, *NOTCH1* and *MYH11* appear to contribute to both BAV development and BAV associated aortopathy. TS DMRs also show TFBS motif enrichment for *TBX20*; copy number variations involving this gene have been identified in BAV subjects with a prevalence ∼1% (MIBAVA Leducq Consortium et al., 2019). *TBX20* is an ancient member of the *TBX* family which has been characterized to be essential for heart development and valvulogenesis in multiple animal models and mutations have been found in congenital heart disease probands (Kirk et al., 2007). Alterations in the function of this transcription factor could lead to heart defects especially in concert with dysregulation of other heart development pathways such as *NOTCH1*. In addition to these findings, the epigenetic regulators *KDM4A* and *HDAC2* were significantly enriched within TS DMRs and these genes have been linked to increased risk of congenital heart disease (Zaidi et al., 2013). Overall, the presence of DMRs within these genes suggest dysregulation of known epigenetic pathways, *TBX20* mediated heart development regulation, and *NOTCH* signaling present in TS which could predispose these individuals to a higher risk of BAV.

Strengths of this study include utilizing a high throughput sequencing approach to analyze DNAm changes and leveraging newly developed allelic methylation analysis techniques to validate biallelic DNAm expression to only analyze TS individuals with a lack of X inactivation within our comparison of interest. Limitations of this study include using whole blood DNA to probe DNAm alterations relevant to the heart in addition to limited study size for each group of interest. The diabetic and lipid status of study participants was not captured at time of enrollment which means we cannot exclude potential confounding due to participants with metabolic disease or dyslipidemia within our study. Diabetes and dyslipidemia could confound this analysis due to potential methylation alterations associated with these diseases being detected by contributing as another unknown source of variation reducing statistical power.

Alternate interpretations of the data include the possibility that having a BAV somehow alters DNA methylation of the blood over time as our study cohort does not include infants. However, the likelihood of BAV having a substantial effect on DNA methylation in remote tissues such of that of the bone marrow (hematopoietic stem progenitors) seems unlikely. We propose that it is more likely that monosomy X leads to genetic and epigenetic dysregulation which causes changes in the canonical cell regulation cascades leading to increased risk of BAV. It is also possible that altered DNA methylation could be a compensatory effect of BAV rather than a risk factor. This study does not have the ability to rule out this possible alternate mechanism. However, the fact that deficiency of a second sex chromosome is a powerful modulator of DNA methylation in general, it is a reasonable premise that specific differences in DNA methylation between individuals with Turner syndrome is a driving force underlying differences in comorbidities such as BAV. Alternate interpretations of the data include the possibility that having a BAV somehow alters DNA methylation of the blood over time as our study cohort does not include infants. However, the likelihood of BAV having a substantial effect on DNA methylation in remote tissues such as bone marrow (hematopoietic stem progenitors) seems unlikely. We propose that it is more likely that monosomy X leads to genetic and epigenetic dysregulation which causes changes in the canonical cell regulation cascades leading to increased risk for BAV. It is also possible that altered DNA methylation could be a compensatory effect of BAV rather than a risk factor. This study does not have the ability to rule out this possible alternate mechanism. However, the fact that deficiency of a second sex chromosome is a powerful modulator of DNA methylation in general, it is a reasonable premise that specific differences in DNA methylation between individuals with Turner syndrome is a driving force underlying differences in comorbidities such as BAV.

Together, these findings suggest that alterations in pathways directed by *TBX20* and *NOTCH1* pathways are altered in TS generally, with BAV individuals also showing DNAm alterations at *PBX3* and *PKNOX1* TFBS. These findings are not powered to distinguish whether these alterations are causal to BAV formation or a downstream effect of the X chromosome monosomy, which leads to BAV. Further studies to validate these findings, as well as functional studies in the appropriate model systems, are needed to elucidate the mechanisms behind the epigenetic basis of BAV formation in TS. Overall, these DNAm changes are most likely due to haploinsufficiency of X escape genes that lead to alterations in epigenetic programming causing the phenotypes associated with TS. This hypothesis is supported by previous epigenetic studies of other sex chromosome abnormalities (47, XXY or 47, XXX) where TS individuals have the largest change in DNAm compared to euploid controls (Trolle et al., 2016; Skakkeb æ k et al., 2018; Zhang et al., 2020). It is important to note that, although X escape genes have been studied for many years, we still lack a complete map of all X escape genes and functional studies of their activity in normal development (Di Palo et al., 2020). Considering the X chromosome has more non-coding RNA than expected and that known genes on the X chromosome have regulatory roles critical to development, there is still much to learn about the function of genes on the X chromosome (Guo et al., 2009; Di Palo et al., 2020).

## Data Availability

The datasets presented in this study can be found in online repositories. The names of the repository/repositories and accession number(s) can be found below: Gene Expression Omnibus, GSE198262.
