# Supplementary material for: DNA Methylation Analysis of Turner Syndrome BAV
Source: Front Genet. 2022 May 31;13:872750. doi: 10.3389/fgene.2022.872750 (PMC9194862; doi:10.3389/fgene.2022.872750)
Supplement: Supplementary file 2 [file DataSheet1.PDF]

Supplemental

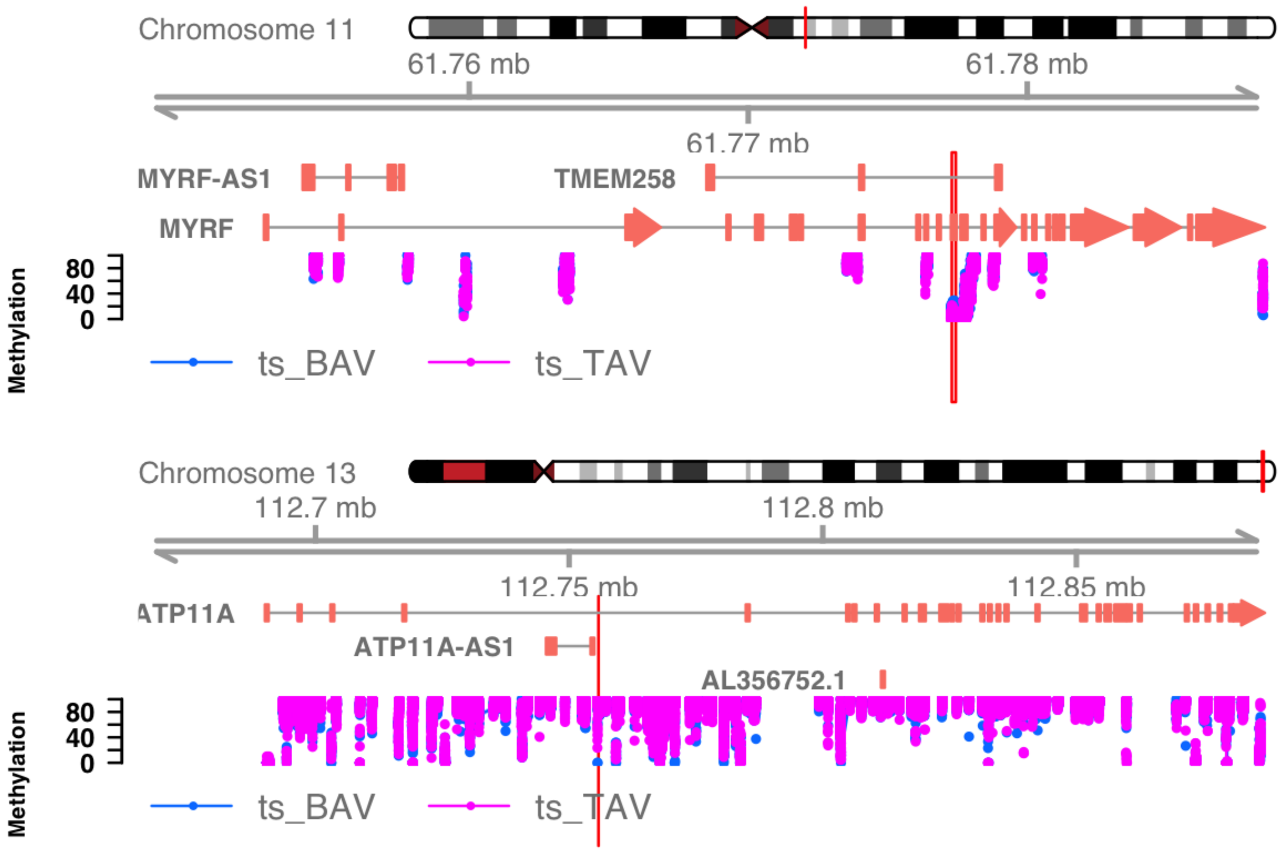


**Supplemental Figure 1: Genome Browser tracks for MYRF and ATP11A.**

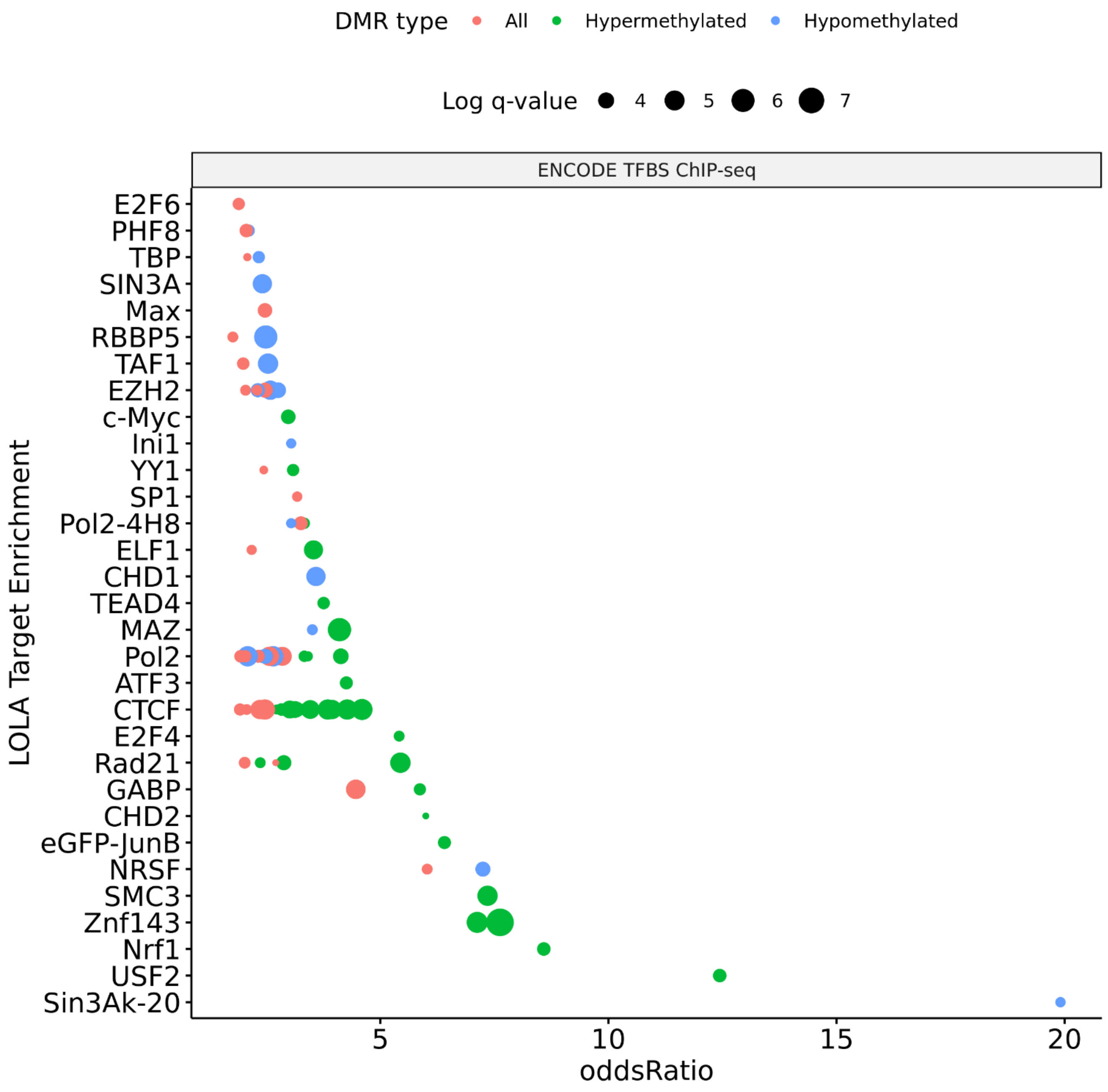


**Supplemental Figure 2: LOLA enrichment analysis for ENCODE TFBS.**

## Supplemental Table 1

## Reactome pathway enrichment results for genes associated with TS BAV DMRs within the promoter region.

| Pathway identifier | Pathway name | #Entities found | #Entities total | Entities ratio | Entities pValue | Entities FDR | #Reactions found | #Reactions total | Reactions ratio | Submitted entities found |
|---|---|---|---|---|---|---|---|---|---|---|
| **R-HSA-164378** | PKA activation in glucagon signalling | 2 | 18 | 0.001551724138 | 7.63E-04 | 0.03730130368 | 1 | 2 | 1.52E-04 | GNAS |
| **R-HSA-392851** | Prostacyclin signalling through prostacyclin receptor | 2 | 20 | 0.001724137931 | 9.40E-04 | 0.03730130368 | 3 | 4 | 3.04E-04 | GNAS |
| **R-HSA-5658208** | Defective SLC5A2 causes renal glucosuria (GLYS1) | 1 | 1 | 8.62E-05 | 0.002238965696 | 0.03730130368 | 1 | 1 | 7.59E-05 | SLC5A2 |
| **R-HSA-8964043** | Plasma lipoprotein clearance | 2 | 33 | 0.002844827586 | 0.002513407196 | 0.03730130368 | 2 | 34 | 0.002580253472 | NR1H2;ACAT2 |
| **R-HSA-163359** | Glucagon signaling in metabolic regulation | 2 | 34 | 0.002931034483 | 0.002664378834 | 0.03730130368 | 4 | 6 | 4.55E-04 | GNAS |
| **R-HSA-420092** | Glucagon-type ligand receptors | 2 | 34 | 0.002931034483 | 0.002664378834 | 0.03730130368 | 1 | 8 | 6.07E-04 | GNAS |
| **R-HSA-381676** | Glucagon-like Peptide-1 (GLP1) regulates insulin secretion | 2 | 43 | 0.003706896552 | 0.004209267705 | 0.04333267168 | 5 | 11 | 8.35E-04 | GNAS |
| **R-HSA-432040** | Vasopressin regulates renal water homeostasis via Aquaporins | 2 | 44 | 0.003793103448 | 0.004401278217 | 0.04333267168 | 5 | 15 | 0.00113834712 | GNAS |
| **R-HSA-382551** | Transport of small molecules | 6 | 731 | 0.06301724138 | 0.004814741298 | 0.04333267168 | 11 | 440 | 0.03339151552 | NR1H2;GNAS;ATP11A;SLC5A2;ACAT2 |
| **R-HSA-418597** | G alpha (z) signalling events | 2 | 49 | 0.004224137931 | 0.005421079825 | 0.0433686386 | 1 | 13 | 9.87E-04 | GNAS |
| **R-HSA-445717** | Aquaporin-mediated transport | 2 | 53 | 0.004568965517 | 0.006307579456 | 0.05046063564 | 5 | 25 | 0.0018972452 | GNAS |
| **R-HSA-9031525** | NR1H2 & NR1H3 regulate gene expression to limit cholesterol uptake | 1 | 5 | 4.31E-04 | 0.01114672226 | 0.06688033359 | 2 | 2 | 1.52E-04 | NR1H2 |
| **R-HSA-9031528** | NR1H2 & NR1H3 regulate gene expression linked to triglyceride lipolysis in adipose | 1 | 5 | 4.31E-04 | 0.01114672226 | 0.06688033359 | 2 | 2 | 1.52E-04 | NR1H2 |
| **R-HSA-174824** | Plasma lipoprotein assembly, remodeling, and clearance | 2 | 72 | 0.006206896552 | 0.01134157045 | 0.06761988386 | 2 | 83 | 0.006298854064 | NR1H2;ACAT2 |
| **R-HSA-422356** | Regulation of insulin secretion | 2 | 79 | 0.006810344828 | 0.01352397677 | 0.06761988386 | 5 | 34 | 0.002580253472 | GNAS |
| **R-HSA-418346** | Platelet homeostasis | 2 | 88 | 0.007586206897 | 0.01657580327 | 0.07957512482 | 3 | 30 | 0.00227669424 | GNAS |
| **R-HSA-201688** | WNT mediated activation of DVL | 1 | 8 | 6.90E-04 | 0.01777730607 | 0.07957512482 | 1 | 4 | 3.04E-04 | CSNK1E |
| **R-HSA-373080** | Class B/2 (Secretin family receptors) | 2 | 97 | 0.008362068966 | 0.0198937812 | 0.07957512482 | 1 | 20 | 0.00151779616 | GNAS |

| | | | | | | | | | | |
|---|---|---|---|---|---|---|---|---|---|---|
| **R-HSA-9029558** | NR1H2 & NR1H3 regulate gene expression linked to lipogenesis | 1 | 9 | 7.76E-04 | 0.019977 98506 | 0.079911 94025 | 8 | 8 | 6.07E-04 | NR1H2 |
| **R-HSA-9623433** | NR1H2 & NR1H3 regulate gene expression to control bile acid homeostasis | 1 | 9 | 7.76E-04 | 0.019977 98506 | 0.079911 94025 | 6 | 6 | 4.55E-04 | NR1H2 |
| **R-HSA-163685** | Integration of energy metabolism | 2 | 109 | 0.009396 551724 | 0.024712 79432 | 0.080545 70553 | 8 | 62 | 0.004705 168096 | GNAS |
| **R-HSA-8866427** | VLDLR internalisation and degradation | 1 | 12 | 0.001034 482759 | 0.026551 61279 | 0.080545 70553 | 1 | 4 | 3.04E-04 | NR1H2 |
| **R-HSA-5610787** | Hedgehog 'off' state | 2 | 114 | 0.009827 586207 | 0.026848 56851 | 0.080545 70553 | 1 | 32 | 0.002428 473856 | GNAS |

## Supplemental Table 2

## Reactome pathway enrichment results for PBX3/PKNOX1 cCRE associated genes.

| Pathway identifier | Pathway name | #Entities found | #Entities total | Entities ratio | Entities pValue | Entities FDR | #Reactions found | #Reactions total | Reactions ratio | Submitted entities found |
|---|---|---|---|---|---|---|---|---|---|---|
| R-HSA-392851 | Prostacyclin signalling through prostacyclin receptor | 2 | 23 | 0.001583803884 | 1.37E-04 | 0.006559635605 | 3 | 4 | 2.98E-04 | GNAS |
| R-HSA-164378 | PKA activation in glucagon signalling | 2 | 23 | 0.001583803884 | 1.37E-04 | 0.006559635605 | 1 | 2 | 1.49E-04 | GNAS |
| R-HSA-420092 | Glucagon-type ligand receptors | 2 | 35 | 0.002410136345 | 3.15E-04 | 0.009850621641 | 1 | 8 | 5.96E-04 | GNAS |
| R-HSA-163359 | Glucagon signaling in metabolic regulation | 2 | 40 | 0.002754441537 | 4.10E-04 | 0.009850621641 | 4 | 6 | 4.47E-04 | GNAS |
| R-HSA-381676 | Glucagon-like Peptide-1 (GLP1) regulates insulin secretion | 2 | 49 | 0.003374190883 | 6.14E-04 | 0.01104347825 | 5 | 11 | 8.19E-04 | GNAS |
| R-HSA-432040 | Vasopressin regulates renal water homeostasis via Aquaporins | 2 | 52 | 0.003580773998 | 6.90E-04 | 0.01104347825 | 5 | 15 | 0.001117235215 | GNAS |
| R-HSA-418597 | G alpha (z) signalling events | 2 | 62 | 0.004269384382 | 9.77E-04 | 0.01270315558 | 1 | 13 | 9.68E-04 | GNAS |
| R-HSA-445717 | Aquaporin-mediated transport | 2 | 68 | 0.004682550613 | 0.001172535829 | 0.01348518359 | 5 | 25 | 0.001862058692 | GNAS |
| R-HSA-8950505 | Gene and protein expression by JAK-STAT signaling after Interleukin-12 stimulation | 2 | 73 | 0.005026855805 | 0.001348518359 | 0.01348518359 | 1 | 36 | 0.002681364517 | HNRNPF |
| R-HSA-9020591 | Interleukin-12 signaling | 2 | 84 | 0.005784327228 | 0.001777444264 | 0.01599699838 | 1 | 56 | 0.00417101147 | HNRNPF |
| R-HSA-447115 | Interleukin-12 family signaling | 2 | 96 | 0.006610659689 | 0.002310078593 | 0.01848062875 | 1 | 114 | 0.008490987636 | HNRNPF |
| R-HSA-373080 | Class B/2 (Secretin family receptors) | 2 | 99 | 0.006817242804 | 0.0024536714 | 0.0196293712 | 1 | 20 | 0.001489646954 | GNAS |
| R-HSA-422356 | Regulation of insulin secretion | 2 | 106 | 0.007299270073 | 0.00280479915 | 0.01963359405 | 5 | 34 | 0.002532399821 | GNAS |
| R-HSA-418346 | Platelet homeostasis | 2 | 117 | 0.008056741496 | 0.003401638102 | 0.02040982861 | 3 | 30 | 0.002234470431 | GNAS |
| R-HSA-5610787 | Hedgehog 'off' state | 2 | 124 | 0.008538768765 | 0.003809814308 | 0.02285888585 | 1 | 32 | 0.002383435126 | GNAS |
| R-HSA-163685 | Integration of energy metabolism | 2 | 145 | 0.009984850572 | 0.005164504784 | 0.03087060681 | 8 | 62 | 0.004617905556 | GNAS |

| | | | | | | | | | | |
|---|---|---|---|---|---|---|---|---|---|---|
| **R-HSA-9660821** | ADORA2B mediated anti-inflammatory cytokines production | 2 | 159 | 0.01094890511 | 0.006174121361 | 0.03087060681 | 5 | 12 | 8.94E-04 | GNAS |
| **R-HSA-5358351** | Signaling by Hedgehog | 2 | 168 | 0.01156865446 | 0.006867280767 | 0.03433640384 | 1 | 82 | 0.00610755251 | GNAS |
| **R-HSA-418555** | G alpha (s) signalling events | 2 | 172 | 0.01184409861 | 0.00718630277 | 0.03593151385 | 10 | 18 | 0.001340682258 | GNAS |
| **R-HSA-5663205** | Infectious disease | 4 | 1343 | 0.0924803746 | 0.01417530853 | 0.05670123412 | 29 | 750 | 0.05586176076 | FXYD4;GNAS;NELFCD |
| **R-HSA-167242** | Abortive elongation of HIV-1 transcript in the absence of Tat | 1 | 27 | 0.001859248037 | 0.02026266076 | 0.05680334617 | 2 | 2 | 1.49E-04 | NELFCD |
| **R-HSA-2022377** | Metabolism of Angiotensinogen to Angiotensins | 1 | 27 | 0.001859248037 | 0.02026266076 | 0.05680334617 | 1 | 20 | 0.001489646954 | CTSZ |
| **R-HSA-9664433** | Leishmania parasite growth and survival | 2 | 297 | 0.02045172841 | 0.02034882726 | 0.05680334617 | 5 | 40 | 0.002979293907 | GNAS |
| **R-HSA-9662851** | Anti-inflammatory response favouring Leishmania parasite infection | 2 | 297 | 0.02045172841 | 0.02034882726 | 0.05680334617 | 5 | 40 | 0.002979293907 | GNAS |
| **R-HSA-6803529** | FGFR2 alternative splicing | 1 | 28 | 0.001928109076 | 0.02100590974 | 0.05680334617 | 3 | 4 | 2.98E-04 | HNRNPF |
| **R-HSA-198933** | Immunoregulatory interactions between a Lymphoid and a non-Lymphoid cell | 2 | 316 | 0.02176008814 | 0.02285563256 | 0.05680334617 | 1 | 44 | 0.003277223298 | SIGLECL1;CD33 |
| **R-HSA-167158** | Formation of the HIV-1 Early Elongation Complex | 1 | 37 | 0.002547858422 | 0.02767212078 | 0.05680334617 | 2 | 5 | 3.72E-04 | NELFCD |
| **R-HSA-113418** | Formation of the Early Elongation Complex | 1 | 37 | 0.002547858422 | 0.02767212078 | 0.05680334617 | 1 | 3 | 2.23E-04 | NELFCD |
| **R-HSA-5694530** | Cargo concentration in the ER | 1 | 37 | 0.002547858422 | 0.02767212078 | 0.05680334617 | 2 | 12 | 8.94E-04 | CTSZ |
| **R-HSA-167243** | Tat-mediated HIV elongation arrest and recovery | 1 | 39 | 0.002685580499 | 0.02914788548 | 0.05680334617 | 3 | 3 | 2.23E-04 | NELFCD |
| **R-HSA-167238** | Pausing and recovery of Tat-mediated HIV elongation | 1 | 39 | 0.002685580499 | 0.02914788548 | 0.05680334617 | 2 | 2 | 1.49E-04 | NELFCD |
| **R-HSA-167287** | HIV elongation arrest and recovery | 1 | 40 | 0.002754441537 | 0.02988500397 | 0.05680334617 | 3 | 3 | 2.23E-04 | NELFCD |
| **R-HSA-167290** | Pausing and recovery of HIV elongation | 1 | 40 | 0.002754441537 | 0.02988500397 | 0.05680334617 | 2 | 2 | 1.49E-04 | NELFCD |
| **R-HSA-382551** | Transport of small molecules | 3 | 958 | 0.06596887481 | 0.03172839373 | 0.05680334617 | 7 | 441 | 0.03284671533 | FXYD4;GNAS |
| **R-HSA-432720** | Lysosome Vesicle Biogenesis | 1 | 43 | 0.002961024652 | 0.03209330784 | 0.05680334617 | 4 | 8 | 5.96E-04 | CTSZ |

| | | | | | | | | | | |
|---|---|---|---|---|---|---|---|---|---|---|
| **R-HSA-9658195** | Leishmania infection | 2 | 406 | 0.0279575816 | 0.03635250679 | 0.05680334617 | 5 | 95 | 0.00707582303 | GNAS |
| **R-HSA-167200** | Formation of HIV-1 elongation complex containing HIV-1 Tat | 1 | 49 | 0.003374190883 | 0.0364962117 | 0.05680334617 | 4 | 5 | 3.72E-04 | NELFCD |
| **R-HSA-168256** | Immune System | 5 | 2681 | 0.184616444 | 0.03690760958 | 0.05680334617 | 6 | 1621 | 0.1207358856 | SIGLECL1;HNRNPF;CTSZ;CD33 |
| **R-HSA-167152** | Formation of HIV elongation complex in the absence of HIV Tat | 1 | 50 | 0.003443051921 | 0.03722825627 | 0.05680334617 | 2 | 2 | 1.49E-04 | NELFCD |
| **R-HSA-167246** | Tat-mediated elongation of the HIV-1 transcript | 1 | 52 | 0.003580773998 | 0.03869082872 | 0.05680334617 | 7 | 8 | 5.96E-04 | NELFCD |
| **R-HSA-167169** | HIV Transcription Elongation | 1 | 52 | 0.003580773998 | 0.03869082872 | 0.05680334617 | 11 | 15 | 0.001117235215 | NELFCD |
| **R-HSA-418594** | G alpha (i) signalling events | 2 | 421 | 0.02899049718 | 0.03884701647 | 0.05680334617 | 1 | 74 | 0.005511693729 | GNAS |
| **R-HSA-112382** | Formation of RNA Pol II elongation complex | 1 | 63 | 0.004338245421 | 0.04669893473 | 0.05680334617 | 2 | 2 | 1.49E-04 | NELFCD |
| **R-HSA-5578775** | Ion homeostasis | 1 | 64 | 0.004407106459 | 0.0474239286 | 0.05680334617 | 2 | 16 | 0.001191717563 | FXYD4 |
| **R-HSA-75955** | RNA Polymerase II Transcription Elongation | 1 | 66 | 0.004544828536 | 0.04887241281 | 0.05680334617 | 6 | 8 | 5.96E-04 | NELFCD |
| **R-HSA-6798695** | Neutrophil degranulation | 2 | 480 | 0.03305329844 | 0.04928355073 | 0.05680334617 | 4 | 10 | 7.45E-04 | CTSZ;CD33 |
| **R-HSA-936837** | Ion transport by P-type ATPases | 1 | 71 | 0.004889133728 | 0.05248486739 | 0.05680334617 | 2 | 15 | 0.001117235215 | FXYD4 |
| **R-HSA-204005** | COPII-mediated vesicle transport | 1 | 77 | 0.005302299959 | 0.05680334617 | 0.05680334617 | 10 | 16 | 0.001191717563 | CTSZ |
| **R-HSA-199992** | trans-Golgi Network Vesicle Budding | 1 | 80 | 0.005508883074 | 0.05895586872 | 0.05895586872 | 4 | 19 | 0.001415164606 | CTSZ |
| **R-HSA-167172** | Transcription of the HIV genome | 1 | 81 | 0.005577744112 | 0.05967238322 | 0.05967238322 | 23 | 47 | 0.003500670341 | NELFCD |
| **R-HSA-9679191** | Potential therapeutics for SARS | 1 | 84 | 0.005784327228 | 0.06181895198 | 0.06181895198 | 1 | 32 | 0.002383435126 | FXYD4 |
| **R-HSA-674695** | RNA Polymerase II Pre-transcription Events | 1 | 88 | 0.006059771381 | 0.06467411414 | 0.06467411414 | 8 | 17 | 0.001266199911 | NELFCD |
| **R-HSA-5654738** | Signaling by FGFR2 | 1 | 88 | 0.006059771381 | 0.06467411414 | 0.06467411414 | 3 | 46 | 0.003426187993 | HNRNPF |
| **R-HSA-6796648** | TP53 Regulates Transcription of DNA Repair Genes | 1 | 89 | 0.00612863242 | 0.06538666932 | 0.06538666932 | 3 | 17 | 0.001266199911 | NELFCD |

| | | | | | | | | | |
|---|---|---|---|---|---|---|---|---|---|
| **R-HSA-500792** | GPCR ligand binding | 2 | 602 | 0.04145434513 | 0.07371566176 | 0.07371566176 | 1 | 185 | 0.01377923432 | GNAS |
| **R-HSA-190236** | Signaling by FGFR | 1 | 107 | 0.007368131111 | 0.07812856681 | 0.07812856681 | 3 | 142 | 0.01057649337 | HNRNPF |
| **R-HSA-449147** | Signaling by Interleukins | 2 | 643 | 0.04427764771 | 0.08268924288 | 0.08268924288 | 1 | 493 | 0.03671979741 | HNRNPF |
| **R-HSA-1643685** | Disease | 4 | 2360 | 0.1625120507 | 0.08862484843 | 0.08862484843 | 29 | 1591 | 0.1185014152 | FXYD4;GNAS;NELFCD |
| **R-HSA-2980736** | Peptide hormone metabolism | 1 | 126 | 0.008676490841 | 0.091406844 | 0.091406844 | 1 | 63 | 0.004692387904 | CTSZ |
| **R-HSA-5576891** | Cardiac conduction | 1 | 138 | 0.009502823303 | 0.09970329298 | 0.09970329298 | 2 | 27 | 0.002011023387 | FXYD4 |
